# The Effect of Supplementation with *Weizmannia coagulans* Strain SANK70258 to Coccidia-Infected Broilers Is Similar to That of a Coccidiostat Administration

**DOI:** 10.3390/vetsci9080406

**Published:** 2022-08-03

**Authors:** Masanori Aida, Ryouichi Yamada, Shin-ichi Nakamura, Taishi Imaoka, Hikari Shimonishi, Toshiki Matsuo, Itaru Taniguchi, Takamitsu Tsukahara

**Affiliations:** 1 Science & Innovation Center, Mitsubishi Chemical Corporation, Yokohama 227-8502, Japan; aida.masanori.ma@m-chemical.co.jp (M.A.); yamada.ryouichi.ms@m-chemical.co.jp (R.Y.); 2 Kyoto Institute of Nutrition & Pathology, Ujitawara 610-0231, Japan; shin-nakamura@ous.ac.jp; 3 KYODOKEN Institute, Kyoto 612-8073, Japan; t-imaoka@kyodoken.co.jp (T.I.); h.shimonishi@kyodoken.co.jp (H.S.); 4 Mitsubishi Chemical Corporation, Tokyo 100-8251, Japan; matsuo.toshiki.mb@m-chemical.co.jp (T.M.); taniguchi.itaru.ma@m-chemical.co.jp (I.T.)

**Keywords:** coccidiosis, *Weizmannia coagulans* strain SANK70258, lasalocid-A sodium, broilers, lesion scores, *Escherichia coli*

## Abstract

**Simple Summary:**

Coccidiosis is caused by protozoan of the genus *Eimeria*, and to control coccidiosis in livestock is crucial for sustainable production. *Eimeria*-infected animals present symptoms such as anorexia, diarrhea, and even death in severe cases. Although the first choice for coccidiosis control is antimicrobial (AM) administration, the emergence of antimicrobial-resistant bacteria and coccidia has become a serious problem and hence AM administration has already been restricted in many countries. Given the above, for coccidiosis control, the development/discovery of compounds in lieu of antimicrobials has been gathering increased attention. Here, we focused on probiotics and examined whether probiotic *Weizmannia coagulans* strain SANK70258 (WC) could be a good alternative to AM to treat *Eimeria*-infected broilers. It was found that WC (1) reduced the numbers of intestinal oocysts; (2) improved intestinal coccidial pathology parameters; and (3) helped increase the body weights of broilers. Furthermore, while intestinal *Escherichia coli* levels, which are associated with intestinal inflammation and weight loss, increased with AM administration, they did not with WC supplementation. Our results suggest that not only is WC a good alternative to AM, but it also treats coccidiosis harmlessly, with no negative effects such as the promotion of potentially pathogenic bacterial growth such as *E. coli*.

**Abstract:**

To determine whether it could also improve the production performance of *Eimeria*-infected broilers, *Weizmannia coagulans* strain SANK70258 (WC) supplementation was compared with coccidiostat lasalocid-A sodium (AM) administration. First, to determine the optimum WC dose, newly hatched broiler chick groups *(n* = 10) were untreated or consecutively given WC (0.005%, 0.01%, 0.03%, and 0.1%) and AM until slaughter (31 days of age). At day 21, all chicks were infected with coccidia. From the economical and practical viewpoints, 0.03% WC supplementation was the best dose. Second, newly hatched broiler chick groups (*n* = 10) were untreated or given 0.03% WC and AM. Each group was run in triplicate. At day 21, two chicks/pen with the farthest body weights as per the group’s mean body weight were spared, and the remaining inoculated with coccidia. At days 42 and 49, the WC and AM groups had significantly greater body weights and daily weight gains. Intestinal lesion scores were lower in 29-day-old AM and WC. Oocyst numbers were lower in 29- and 49-day-old AM and WC, but only 29- and 49-day-old AM had higher *Escherichia coli* levels. To conclude, although WC and AM induced similar growth performance in coccidium-infected chicks, unlike AM, the *E. coli* levels did not increase with WC.

## 1. Introduction

Coccidiosis in broilers is caused by parasites of the apicomplexan genus *Eimeria* [1]. Coccidiosis causes, in birds, low utilization of feed, bloody diarrhea, and lower immune response to enteric diseases, which leads to low feed conversion ratio, undergrowth, and high mortality [2,3]. In 2016 alone, the losses that the U.S.A. and Brazil, the world’s largest broiler producers, incurred due to coccidiosis infection were estimated to be GBP 1175.88 million and GBP 958.62 million, respectively [4].

Although recent studies have alerted and raised concern about the rapid development of antimicrobial resistance in *Eimeria* spp. [5], anticoccidial drugs have been the gold standard for the treatment of coccidiosis in broilers in the past decades [6]. Therefore, the need for the development and/or discovery of alternative compounds to antimicrobials to fight coccidiosis has been increasingly voiced by scientific communities.

Probiotics is a generic term used to refer to those microorganisms that have been shown to confer health properties to the host upon their consumption. Probiotics have been recently used to mitigate and/or treat the effects of infection with parasites such as *Trichinella* spp., *Toxoplasma gondii*, helminths such as *Ascaris* spp., and *Parascaris* spp. as well as *Eimeria* spp. [2,3,7,8,9,10,11,12,13]. For example, Mohsin et al. demonstrated that when *Eimeria tenella*-infected chickens were supplemented with *Lactobacillus plantarum*, their cell-mediated and humoral immune responses increased, and the activity of antioxidant enzymes superoxide dismutase 1 and catalase, peptide transporter 1, and tight junction proteins zonula occludins and claudin 1 improved [2]. Similarly, Wang et al. gave low (1 × 10^7^ CFU/g of feed) and high (1 × 10^8^ CFU/g of feed) doses of *L. plantarum* supplementation, in unison with the coccidiostat Diclazuril, to broilers infected with *Eimeria* [12]. Unlike the control broilers, whose mortality and oocyst shedding increased, those receiving *L. plantarum* supplementation and the coccidiostat had fewer deaths, less oocyst shedding, a greater growth performance, and improved intestinal barrier and morphology as well as reduced inflammation parameters [12]. Separately, past work showed that supplementation to broilers with 5 × 10^9^ CFU of *Lactobacillus sporogenes* [14,15], also known as *Bacillus coagulans* [14] or *Weizmannia coagulans* [16], per kg of feed, resulted in the supplemented birds having greater body weights and average daily gains [17]. *W. coagulans* also helped to reduce the serum levels of proinflammatory factors and the growth of potentially pathogenic *Parasutterella*, while at the same time inducing a greater production of short-chain fatty acids (SCFA) and the growth of beneficial bacteria *Alistipes* and *Odoribacter* [17]. Likewise, supplementation of 1 × 10^9^ spores of *W. coagulans* strain SANK70258 (WC) per gram of a concoction (100 ppm) to White Leghorn cockerels, helped increase the body weights and feed efficiency of the birds [15]. In an in vitro study, Sasaki et al. co-cultured human colonic microbiota (Kobe University Human Intestinal Microbiota Model or KUHIMM) with WC [18]. They found that at a concentration of 4 × 10^7^ of total cells/mL of culturing medium, the growth of potentially pathogenic *Enterobacteriaceae* including *E. coli* was suppressed while that of SCFA-producing *Lachnospiraceae* was induced.

In the present study, to determine whether it could also promote the production performance in broilers by suppressing the pathological and microbiological parameters, we aimed to compare the probiotic *W. coagulans* strain SANK70258 (WC) with coccidiostat lasalocid-A sodium (AM). The *E. coli* levels in the intestines of broilers given WC and AM were also investigated because in coccidium-infected broilers, indigenous *E. coli* population is predominant [19], which is known to induce inflammation by stimulating the lipopolysaccharide production [20].

## 2. Materials and Methods

### 2.1. Experiment 1

Experiment 1 was conducted to explore the necessary percentage of WC in a probiotic product needed to confer health benefits to coccidium-infected broilers. The experimental design and sampling procedure are summarized in Figure S1.

#### 2.1.1. Preparation of Probiotic

The probiotic *W. coagulans* SANK70258 manufactured by Mitsubishi Chemical (Tokyo, Japan) containing 1 × 10^9^ CFU per g was used. Supplementation was given to broiler groups at 0.005, 0.01, 0.03, and 0.10% (*w*/*w*), so that diets contained 5 × 10^4^ CFU, 1 × 10^5^ CFU, 3 × 10^5^ CFU, and 1 × 10^6^ CFU per g of feed.

#### 2.1.2. Animals and Diets

Sixty newly hatched Ross308 broiler chicks (mean body weight: 37 g) were purchased from a commercial supplier and kept in a closed shed at the KYODOKEN Institute (Kyoto, Japan). They were equally divided into the following groups (*n* = 10; five males and five females) with comparable body weights: treatment-free control (C), 0.005% WC supplementation (WC_0.005), 0.01% WC supplementation (WC_0.01), 0.03% WC supplementation (WC_0.03), 0.1% WC supplementation (WC_0.1), and sodium lasalocid-A administration at 75 ppm (A). Each chick group was housed in a floored-pen (W: 0.80 × D:1.20 × H: 0.45 m) with sawdust bedding and equipped with a gas brooder and a heat lamp to maintain an adequate, stable environment. Feed and drinking water were supplied ad libitum. The diet of broilers was formulated by the Japan Scientific Feeds Association (Chiba, Japan). The composition and nutrient content (%) of the diet is shown in Table S1. The diet was given to broilers from 0 to 31 days of age. All animals used in the present study were identified with color markers, and individually weighed at 0, 21, and 31 days of age. In addition, the feed intake in each pen was calculated on the same days.

The animals in the present study were handled in accordance with the guidelines for animal studies of the KYODOKEN Institute (approval number ET194021).

#### 2.1.3. Coccidial Challenge

For the experimental infection in Experiment 1, clinical *Eimeria* strains isolated from broiler farms were used. A suspension containing *E. tenella* and *Eimeria acervulina* was prepared so that it contained 5000 and 100,000 oocysts of the respective strains per mL. One mL of the suspension was orally given to each bird at 21 days of age.

#### 2.1.4. Dissection and Sampling Procedure

At 31 days of age, all chicks were dissected. The slaughter day (day 31) was decided as per the life cycle of *E. tenella*. Broilers were euthanized by exsanguination under deep sedation with intravenous injections of a sodium pentobarbital saline solution (64.8 mg/mL; pentobarbital sodium salt, Nacalai tesque, Kyoto, Japan). Upon celiotomy, the intestines of the broilers were removed, and the intestinal lesion scores were determined as previously described [21]. The intestines were separated into the small intestines and the ceca, and their lengths were measured. Next, the small intestinal and cecal digestas were aseptically collected into sterile tubes, and immediately stored at 4 °C.

#### 2.1.5. Coccidial Count in Intestinal Digesta

The numbers of oocysts in the cecal digestas were determined using a McMasters counting chamber. Small (*E. acervulina*), middle (*E. tenella*), or large (other coccidia such as *E. maxima*) oocysts in the digestas were counted using a light-microscopy.

#### 2.1.6. Statistical Analysis

Depending on the results of the Bartlett test, either a completely randomized design one-way analysis of variance or the Kruskal–Wallis test was used to analyze the differences between the means of the body weights, oocyst numbers, and the lengths of the small intestines (*n* = 10). Either Dunnett’s or Steel’s post hoc comparison was used for multiple comparisons, as needed. The Kruskal–Wallis test was used to analyze the differences in the lesion scores of the intestines (*n* = 10). Again, Steel’s post hoc comparison was used for multiple comparisons, as needed. In all of the statistical analyses, the values were the means and standard deviations. In addition, the differences between the means were considered significant if *p* < 0.05. All statistical analyses were conducted using R software version 4.1.2 (R Core Team, Vienna, Austria).

### 2.2. Experiment 2

#### 2.2.1. Preparation of the Probiotic

Probiotic *W. coagulans* SANK70258, manufactured by Mitsubishi Chemical and containing 1 × 10^9^ CFU per g, was used. Based on that, the diet of the broiler 0.03% (*w*/*w*) treatment group contained 3 × 10^5^ CFU of the probiotic/g of feed.

#### 2.2.2. Birds and Diets

Ninety newly hatched male Ross308 broiler chicks (mean body weight, 42 g) were purchased from a commercial supplier. The broiler chicks were equally divided into three groups (*n* = 10) with comparable body weights: control, WC supplementation (WC), and the administration of 75 ppm of the antibacterial agent and coccidiostat lasalocid-A sodium (AM). The experimental broiler groups were run in triplicate and kept in separate floor-pens (W: 0.80 × D: 1.20 × H: 0.45 m) in a shed at the KYODOKEN Institute (Kyoto, Japan). The pens were furnished with sawdust bedding, gas brooders, and heat lamps to maintain an adequate, stable environment. Feed and drinking water were supplied ad libitum. The experimental diets were formulated by the Japan Scientific Feeds Association (Chiba, Japan). The compositions and nutrient contents of the diets are shown in Table S2. The starter diet was fed to broiler chicks from 0 to 21 days of age, and the grower diet from 21 to 49 days of age. All animals used in the present study were identified by color markers and individually weighed at 0, 7, 14, 21, 28, 35, 42, and 49 days of age (49 days of age is a common rearing period in Japan). The feed intake per pen was also measured on the same days. The broiler chicks were handled and cared for as per the guidelines for animal studies of the KYODOKEN Institute (approval number EB214027). The experimental design and sampling procedure are summarized in Figure S2.

#### 2.2.3. Coccidial Challenge

At 21 days of age, eight chicks per pen were selected as per the mean body weight in each pen and the remaining two were spared from the experiment.

Clinical strains of *E. tenella* and *E. acervulina*, isolated from broiler farms (same strains as those used in the Experiment 1), were used for the experimental infection as follows. Equal volumes of suspensions containing 5000 and 100,000 oocysts of *E. tenella* and *E. acervulina*, respectively, were mixed to produce a final suspension of 1 mL, which was orally given at 21 days of age to every chick in the pens (*n* = 8).

#### 2.2.4. Dissection and Sampling Procedure

Three chicks were selected for dissection at 29 days of age, as per the mean body weight in each pen. The slaughter day (day 29) was decided as per the life cycle of *E. tenella* and *E. acervulina*. Broiler chicks were euthanized by exsanguination under deep sedation with intravenous injections of a sodium pentobarbital saline solution (64.8 mg/mL; pentobarbital sodium salt, Nacalai tesque, Kyoto, Japan). Upon celiotomy, the intestines of the broilers were removed, and the intestinal lesion scores were determined as previously described [21]. The small intestines and the ceca were removed from the intestines, and their lengths were measured. Next, the small intestinal and cecal digestas were aseptically collected into sterile tubes, and immediately stored at 4 °C. Portions of the duodenal (immediately before the U-turn point) and ileal (terminal portions of the small intestines) tissues were soaked into an RNA-later solution (Sigma-Aldrich, Tokyo, Japan). In addition, separate portions of the duodena, the ilea, and the ceca were fixed with a 10% (*v*/*v*) phosphate-buffered formalin solution. The intestinal tissues soaked in RNA-later solution were stored at −80 °C and used for a separate study.

Following the dissection of all chicks and to conduct coccidial counts and bacteriological studies, the digestas were divided into two portions. The portions used for the coccidial counts were continuously stored at 4 °C, whereas the portions used for the bacteriological studies were stored at −80 °C.

The remaining five chicks in each pen were raised until they reached 49 days of age, at which three chicks with body weights similar to the mean body weight/pen were dissected as described above.

#### 2.2.5. Coccidial Count in Intestinal Digesta

The numbers of oocysts per gram (OPG) in the small intestinal and cecal digestas were determined using a McMasters counting chamber. Small (*E. acervulina*), middle (*E. tenella*), and large (other coccidia such as *E. maxima*) oocysts in the digestas were counted using light microscopy.

#### 2.2.6. Bacteriological Analysis

The small intestinal and cecal digestas were used for bacteriological analysis by real-time PCR, as previously described [22]. The primers and the PCR conditions for the analysis of total bacteria and *E. coli* DNA are shown in Table S3. For each aforementioned reaction, the positive control and the negative water control were assayed together with the samples. To verify the specificity of the reaction, the melting curves of the amplified DNA were generated.

#### 2.2.7. Histopathologic Observation

Fixed intestines were embedded in paraffin wax and cut into 4-μm-thick serial paraffin sections. These paraffin sections were then stained with hematoxylin–eosin to measure the height of intestinal villi and the depth of the intestinal crypts using light microscopy, as previously described [23].

#### 2.2.8. Statistical Analysis

Depending on the results from the Bartlett test, either a completely randomized design one-way analysis of variance or the Kruskal–Wallis test was used to analyze differences between the parameters of the growth performance (body weight, daily weight gain, and feed conversion ratio; *n* = 3), the body weight of dissected chicks, oocyst, and bacterial numbers, and the length of the intestines (*n* = 9). Dunnett’s or Steel’s post hoc comparisons were used for multiple comparisons, as needed. The Kruskal–Wallis test was used to analyze the differences between the lesion scores of the intestines (*n* = 9). Again, Steel’s post hoc comparisons were used for multiple comparisons, as needed. At 29 and 49 days of age, the correlation coefficients between the parameters of the growth performance (body weight) and the coccidiosis status (oocyst numbers and lesion scores of the small intestines), respectively, were further analyzed by the Pearson product–moment correlation coefficient.

In all of the statistical analyses, the values were the means and standard deviations. In addition, the differences between the means were considered significant if *p* < 0.05, and with a tendency to be significant if *p* < 0.1. All statistical analyses were conducted using R software version 4.1.2 (R Core Team, Vienna, Austria).

## 3. Results

### 3.1. Experiment 1

Experiment 1 (WC supplementation at different levels) was considered as an exploratory experiment. The results of the effect of WC supplementation on the coccidial infection of broilers are shown in Table 1. Parameters such as body weights, lesion scores, and intestinal lengths changed little between broiler groups (*p* > 0.05). The higher the concentration of the WC supplementation, the lower the number of oocysts observed (*p* = 0.104). Moreover, between days 0 and 31, the feed conversion ratios (FCR) between the control (1.86), WC_0.005 (1.81), and WC_0.01 (1.82) groups changed little. Nonetheless, the FCR for WC_0.03 and WC_0.1 were 1.66 and 1.52, respectively, which were considered as promising results. However, for economical and practical purposes, only a WC supplementation at a level of 0.03% was used in Experiment 2.

### 3.2. Experiment 2

#### 3.2.1. Effect of WC Supplementation on Production Performance of Coccidiosis Broilers

**Figure 1:**
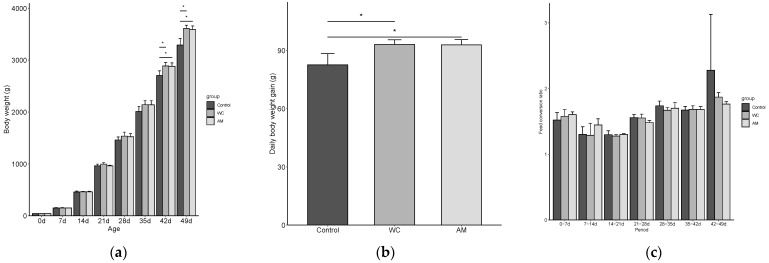
The production performance parameters of broilers, pre- (0 to 21 days of age) and post- (21 to 49 days of age) coccidial infection, in Experiment 2. (**a**) Mean body weights throughout the experiment. (**b**) Daily weight gains post-coccidial infection. (**c**) Feed conversion ratios throughout the experiment. Bars represent the means of data per pens and error bars represent the standard deviations (*n* = 3). WC, supplementation with 3 × 10^5^ CFU of *Weizmannia coagulans* strain SANK70258/g; AM, 75 ppm of sodium lasalocid-A in feed. Asterisks indicate the significant difference between groups (*p* < 0.05).

From day 28 (broiler’s age) onward and compared with those of the control, the body weights of the WC and AM tended to be non-statistically higher, but became significantly greater between days 42 (*p* = 0.042) and 49 (*p* = 0.009) (Figure 1a). The average daily weight gains of AM and WC between days 21 and 49 (post-coccidial infection) were significantly higher (*p* = 0.026) when compared with those of the control (Figure 1b). In addition, between days 42 and 49, the AM and WC tended to have non-statistically lower FCR than the control (*p* > 0.05) (Figure 1c).

#### 3.2.2. Effect of WC Supplementation on Intestinal Lesion Scores and Number of Oocysts of Coccidiosis Broilers

The body weights of all chicks slaughtered on day 29 (three per pen, nine per group) were compared. While no significant differences were observed between the AM and the control, the WC had greater body weights than the control (*p* = 0.054). At day 29 and when compared with the control, the lesion scores were found to be lower (*p* = 0.001) in the small intestinal samples of AM and WC (Table 2), but by day 49, no significant differences were observed (Table 3) between the samples (*p* = 0.489). At day 29 and when compared with the control, the number of oocysts was significantly lower and tended to be lower in the small intestinal digestas of AM and WC, respectively (*p* < 0.001). At day 49, the number of oocysts in the small intestinal digestas was significantly lower in the AM and WC than in the control (*p =* 0.002). With respect to the number of oocysts in the cecal digestas, while no differences in the counts were observed between groups at day 29 (*p* = 0.554), the numbers of oocysts in the cecal digesta of 49-day-old broilers were found to be significantly lower in the AM and WC, when compared with the control (*p* = 0.033).

**Figure 2:**
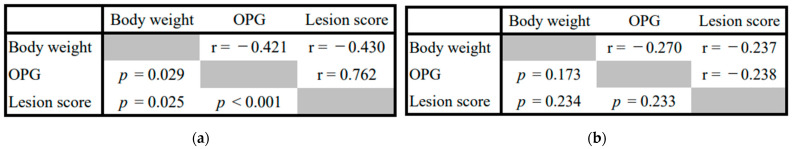
The Pearson product–moment correlation coefficient between the body weights, number of oocysts (OPG), and intestinal lesion scores of the coccidiosis-infected broilers. The *p* values are shown in the lower left and the rho values in the upper right. (**a**) Twenty-nine days of age; (**b**) Forty-nine days of age.

For the 29-days-old broilers, the correlations between the numbers of OPG, lesion scores, and body weights were significantly negative (r = −0.421, *p* = 0.029, and r = −0.430, *p* = 0.025, respectively), whereas the correlation between the lesion scores and the number of OPG was significantly positive (r = 0.762, *p* < 0.001). For the 49-day-old broilers, the correlations between these parameters were non-significant. (Figure 2).

#### 3.2.3. Effect of WC Supplementation on Total Bacteria and *Escherichia coli* Counts in Digesta Samples of Coccidiosis Broilers

At 29 days of age and when compared with the control, the total bacterial counts were lower in both the WC and AM (*p* < 0.001), but only lower in AM at 49 days of age (*p* < 0.001). Of these bacteria, the *E. coli* levels were considered crucial due to their potential detrimental activity on the growth performance. At 29 and 49 days of age and when compared with the control, the percentages of *E. coli* were significantly higher in AM, but those of WC and the control remained unchanged (*p* < 0.001 and *p* = 0.004, respectively) (Table 2 and Table 3).

#### 3.2.4. The Effect of WC Supplementation on the Intestinal Morphology of Coccidiosis Broilers

No significant differences were found between the lengths of neither the small intestines nor the ceca of the broilers (*p* > 0.05) (Table 2 and Table 3).

## 4. Discussion

As the standard procedure, antimicrobials such as coccidiostats have been used to treat coccidiosis in poultry [5]. However, increased drug resistance in *Eimeria* spp. has prompted researchers to find alternative compounds to traditional treatments. Probiotics have emerged as serious and viable alternatives to drugs for the treatment of coccidiosis in broilers [2,12]. In the present work, we wanted to evaluate whether WC exerted an effect on coccidiosis as effective as that of coccidiostat AM.

In the present study, parameters such as body weight, weight gain, number of OPG, and lesion scores were used to evaluate the therapeutic effect of WC supplementation. The number of OPG was significantly lower or tended to be lower in AM and WC than in the control. Moreover, as lesion scores and the number of OPG decreased, the body weights of the broilers increased (Figure 2). These negative correlations seemed to indicate that lower intestinal numbers of OPG likely contributed to greater body gains. While lower numbers of OPG were somewhat expected upon the administration of AM, given the documented efficacy of lasalocid-A sodium to treat coccidiosis in livestock [24,25], the count-lowering effect of WC on oocysts was a head-turning outcome. *L. plantarum* supplementation was reported to help decrease the number of OPG in the feces of coccidia-infected chicken [2], but to the best of our knowledge, our work is the first to report the effectiveness of *W. coagulans* supplementation to help decrease the number of OPG in broilers. In the present study, while the underlying mechanism by which WC supplementation suppressed the numbers of OPG in the digestas of broilers remained unclear, two hypotheses were plausible. First, bacteriocin produced by WC helped decrease the numbers of OPG. Past studies have reported that bacteriocin produced by beneficial bacteria had an anticoccidial effect [26,27]. Furthermore, Hyronimus et al. reported that WC produced a bacteriocin-like inhibitory substance [28], which may have a similar inhibitory effect as those previously reported by Hessenberger et al. [26] and Pogány Simonová et al. [27]. Second, the production of *Eimeria*-specific antibodies was induced by WC supplementation. Lee et al. supplemented coccidium-infected broilers with probiotics *Pediococcus acidilactici* and *Saccharomyces boulardii* [29]. These authors observed that the blood sera of probiotic-supplemented broilers had high levels of *Eimeria*-specific antibodies [29]. In addition, Zhang et al. and Zhen et al. found that WC supplementation increased the level of immunoglobulin (Ig) A in the feces of healthy [17] and *Salmonella* Enteritidis-infected broilers [30], respectively. IgA is an important immune factor in animals, contributing to the immune defense in vivo [17]. We believe that in the present work, the IgA production was induced by WC supplementation, which may have functioned as an adjuvant that enhanced the immune response during coccidiosis. An enhanced immune response likely helped WC and AM gain more weight than the control, as nutrient absorption and utilization shifted from immune cell production back to growth performance [2], which agrees with the results reported by Zhang et al. [17].

In the present study, it was found that WC supplementation significantly lowered the small intestinal lesion scores of broilers at 29 days of age, but by 49 days of age, no differences were observed between the broiler groups. It is possible that by 49 days of age, the acquired immunity in all broilers including the control was prompted into action, which helped the birds recover from coccidial symptoms. Therefore, we believe that WC supplementation may be more effective if given during the early stage of coccidial infection, when inflammation is a major symptom. Past studies have shown that *W. coagulans* contributes to reduce intestinal inflammation by modulating the intestinal cytokine profiles of mice [31,32]. In broilers, *W. coagulans* supplementation exerts an anti-inflammatory effect by inducing the downregulation of the expression of proinflammatory cytokine IFNγ [33]. Cytokine IFNγ has been singled out as being one of the factors that increase the gut lesion scores in broilers infected with necrotic enteritis-causing *Clostridium perfringens* [33]. Similarly, Yu et al. demonstrated that *W. coagulans* supplementation exerted an anti-inflammatory effect on lipopolysaccharide-induced systemic inflammation by decreasing the levels of pro-inflammatory factors TNF-α, IL-1β, IL-6, and IFN-β, while increasing that of the anti-inflammatory factor IL-10 in serum and jejunal mucosae of broilers [20]. Separately, Chaudhari et al. challenged broilers with several strains of *Eimeria* spp. and afterward, supplemented them with probiotic bacilli [34]. They found that, compared with the control, probiotic-supplemented broilers had lower intestinal lesions, which was correlated with the increase in the expression of anti-inflammatory molecules IL-10 and TGF-β [34]. These reports seem to suggest that by inducing the production of anti-inflammatory molecules, *Bacillus* supplementation modulated the innate immune response, which protected the intestine of the pathogen-infected host from damage by its own immune system. Thus, we believe that, in the present study, WC played a similar immunomodulatory role. It is recommended that the profiles of intestinal cytokines and their immunomodulation functions be further investigated.

As it has been previously reported that not only the information on the numbers of individual bacterial communities but also the information on the ratio between them are crucial when analyzing the gut microbiota [35], in the present study, the total bacterial counts and *E. coli* levels in relation to the total bacterial counts were also calculated. When compared with those of the control, at 29 days of age, the total bacterial counts were lower in the intestinal digestas of both the WC and AM (Table 3), whereas at 49 days of age, the total bacterial count was lower only in AM, even though lasalocid-A sodium had already been withdrawn at 42 days of age. Furthermore, at 29 and 49 days of age and when compared with the other broiler groups, the number of *E. coli* increased only in AM. It is well-known that antimicrobials exert detrimental effects on the health, the bacterial count, and diversity of the gut microbiota of broilers and can even cause the development of drug resistance in pathogens, if misused or overused [34]. Indeed, the dysbiosis of the microbiota caused by antimicrobials can be long-lasting, even after discontinuation of the antimicrobial treatment [36]. For example, monensin, an ionophore therapeutic drug similar to lasalocid-A sodium, has been reported to increase the *Escherichia*/*Shigella* abundance [37], which is consistent with our results, showing an increase of *E. coli* in AM. Since an increased number of *E. coli* induces the production of inflammation-causing lipopolysaccharides in broilers [20], it can be inferred that the use of lasalocid-A sodium to treat broilers is not always beneficial. As our results indicate that WC supplementation can modulate the intestinal microbiota in coccidium-infected broilers, we plan to use next-generation sequencing to further analyze the microbiota compositions of broilers in future work.

Upon the measurement of the intestinal lengths, it was observed that those of the WC tended to be longer than those of the control and AM, which could be attributed to the therapeutic effects of WC, which in turn led to greater body weights (Figure 1b) and perhaps better nutrient absorption [38].

## 5. Conclusions

The present study had some limitations. For example, the number of experimental animals and replicability were limited. In addition, the mechanism by which WC suppressed the numbers of OPG in the digestas remained unclear. These limitations need further exploration. Nonetheless, we demonstrated the suitability of WC as a probiotic to treat the adverse effects of coccidiosis in broilers. Both WC supplementation and lasalocid-A sodium administration induced similar body weights and average daily weight gains. In addition, WC supplementation decreased the lesion scores and OPG numbers in a greater manner than the control and only better ranked by AM. Finally, unlike lasalocid-A sodium, WC supplementation did not seem to adversely alter the microbiota of the coccidiosis-infected broilers.

## Figures and Tables

**Table 1:** The body weights, feed conversion ratios, lesion scores, oocyst numbers in the cecum, and in the intestinal lengths of broiler chicks in Experiment 1.

Parameter	C	WC_0.005	WC_0.01	WC_0.03	WC_0.1	A	*p* Value ^†^
Body weight (g)							
Day 0	37 ± 2	37 ± 2	36 ± 1	36 ± 2	37 ± 2	37 ± 2	0.990
Day 21	795 ± 114	812 ± 99	810 ± 105	864 ± 135	870 ± 74	849 ± 81	0.480
Day 31	1657 ± 205	1698 ± 196	1670 ± 176	1697 ± 223	1774 ± 159	1740 ± 171	0.768
Feed conversion ratio (g/g)							
Day 0–21	1.87	1.87	1.86	1.79	1.63	1.90	–
Day 21–31	1.84	1.76	1.79	1.57	1.43	1.65	–
Day 0–31	1.86	1.81	1.82	1.66	1.52	1.76	–
Lesion score (score 0–4 ^‡^)							
Small intestine	1.5 ± 0.6	1.4 ± 0.6	1.6 ± 0.7	1.1 ± 0.4	1.6 ± 0.6	1.3 ± 0.5	0.298
Cecum	1.5 ± 0.6	1.4 ± 0.6	1.1 ± 0.6	1.3 ± 0.5	1.2 ± 0.4	1.1 ± 1.4	0.720
Oocyst numbers in the cecum (×10^4^ oocyst/g)	8.66 ± 1.79	7.69 ± 0.88	6.87 ± 0.78	6.86 ± 1.96	6.64 ± 1.20	9.92 ± 6.34	0.104
Intestinal length (cm)							
Small intestine	154.6 ± 10.8	161.5 ± 16.3	175.9 ± 18.3	163.9 ± 20.3	165.3 ± 16.4	164.6 ± 17.3	0.154
Cecum	16.3 ± 2.2	16.4 ± 3.1	16.4 ± 2.5	16.0 ± 2.4	17.0 ± 1.8	17.9 ± 1.8	0.504

Except for the feed conversion ratios, all parameters are shown as the means ± standard deviations (*n* = 10). Feed conversion ratios are shown as single values because broiler chicks of a same group were allocated together into pens. C, non-treatment control; WC_0.005, supplementation with 5 × 10^4^ CFU of *Weizmannia coagulans* strain SANK70258/g; WC_0.01, 1 × 10^5^ CFU/g; WC_0.03, 3 × 10^5^ CFU/g; WC_0.1, 1 × 10^6^ CFU/g of feed; A, 75 ppm of sodium lasalocid-A in feed. Days indicate the ages of broiler chicks. ^†^ Probability value of one-way analysis of variance or the Kruskal–Wallis test. ^‡^ The criteria for the lesion scores were as described by Johnson and Reid [21].

**Table 2:** The body weights, lesion scores, oocyst numbers, total bacterial counts, *E. coli* levels, and intestinal lengths of broiler chicks slaughtered at 29 days of age.

Parameter	Control	WC	AM	*p* Value ^‡^
Body weight (g)	1502 ± 87	1596 ± 92 *	1579 ± 69	0.054
Lesion score (score 0–4 ^§^)				
Small intestine	1.67 ± 0.71	0.89 ± 0.33 *	0.67 ± 0.50 *	0.001
Cecum	0.89 ± 0.60	0.67 ± 0.50	0.67 ± 0.50	0.603
Oocyst numbers (oocyst/g)				
Small intestinal contents	8511 ± 1609	6478 ± 1944 ^†^	5644 ± 716 *	<0.001
Cecum contents	2889 ± 909	2533 ± 781	2478 ± 342	0.554
Total bacterial counts (log cell/g)	10.5 ± 0.1	10.3 ± 0.1 *	10.3 ± 0.2 *	<0.001
*E. coli* levels				
Counts (log cell/g)	6.65 ± 0.42	6.85 ± 0.49	7.56 ± 0.38 *	<0.001
Ratio (%)	0.02 ± 0.02	0.07 ± 0.09	0.27 ± 0.18 *	<0.001
Intestinal length (cm)				
Small intestine	152 ± 16	155 ± 15	154 ± 19	0.924
Cecum	12.0 ± 1.5	12.2 ± 1.4	12.9 ± 2.2	0.503

All parameters are shown as the means ± standard deviations (*n* = 9). Control, non-treatment control; WC, supplementation with 3 × 10^5^ CFU/g of *W. coagulans* strain SANK70258; AM, 75 ppm of sodium lasalocid-A in feed. The asterisks indicate significant differences between the control and WC or AM (*p* < 0.05). A dagger indicates a differential trend between the control and WC or AM (*p* < 0.1). ^‡^ Probability value of the one-way analysis of variance or the Kruskal–Wallis test. ^§^ The criteria for the lesion scores were as described by Johnson and Reid [21].

**Table 3:** The body weights, lesion scores, oocyst numbers, total bacterial counts, *E. coli* levels, and intestinal lengths of the broiler chicks slaughtered at 49 days of age.

Parameters	Control	WC	AM	*p* Value ^‡^
Body weight (g)	3316 ± 291	3538 ± 228	3574 ± 261	0.096
Lesion score (score 0–4 ^§^)				
Small intestine	1.22 ± 0.44	0.89 ± 0.60	1.00 ± 0.71	0.489
Cecum	0.33 ± 0.50	0.33 ± 0.50	0.11 ± 0.33	0.494
Oocyst numbers (oocyst/g)				
Small intestinal contents	5867 ± 660	5100 ± 684 *	4700 ± 522 *	0.002
Cecum contents	1922 ± 156	1567 ± 387 *	1578 ± 323 *	0.033
Total bacterial counts (log cell/g)	10.6 ± 0.2	10.5 ± 0.1	10.2 ± 0.3 *	<0.001
*E. coli* levels				
Counts (log cell/g)	6.16 ± 0.48	5.90 ± 0.52	6.40 ± 0.44 *	0.106
Ratio (%)	0.01 ± 0.00	0.00 ± 0.00	0.03 ± 0.02 *	0.004
Intestinal length (cm)				
Small intestine	155 ± 10	165 ± 8	158 ± 14	0.139
Cecum	15.4 ± 1.7	15.8 ± 2.0	16.6 ± 1.9	0.460

The asterisks indicate significant differences between the control and WC or AM (*p* < 0.05). ^‡^ Probability value of one-way analysis of variance or the Kruskal–Wallis test. ^§^ The criteria for the lesion scores were as described by Johnson and Reid [21]. Further information can be found in Table 2.

## Data Availability

Data are available by the corresponding author.

## Supplementary Materials

The following supporting information can be downloaded at: https://www.mdpi.com/article/10.3390/vetsci9080406/s1, Table S1: The feed composition and nutrient content of the diet in Experiment 1; Table S2: The feed composition and nutrient content of the experimental diets in Experiment 2; Table S3: Primers and PCR cycle conditions used in Experiment 2 [39,40]; Figure S1: The experimental design and sampling procedure in Experiment 1; Figure S2: The experimental design and sampling procedure in Experiment 2.
