# Supplementary material for: The Effect of Supplementation with Weizmannia coagulans Strain SANK70258 to Coccidia-Infected Broilers Is Similar to That of a Coccidiostat Administration
Source: Vet Sci. 2022 Aug 3;9(8):406. doi: 10.3390/vetsci9080406 (PMC9416079; doi:10.3390/vetsci9080406)
Supplement: Supplementary file 1 [file vetsci-09-00406-s001.zip › Supplementary Figures S1 and S2.pdf]

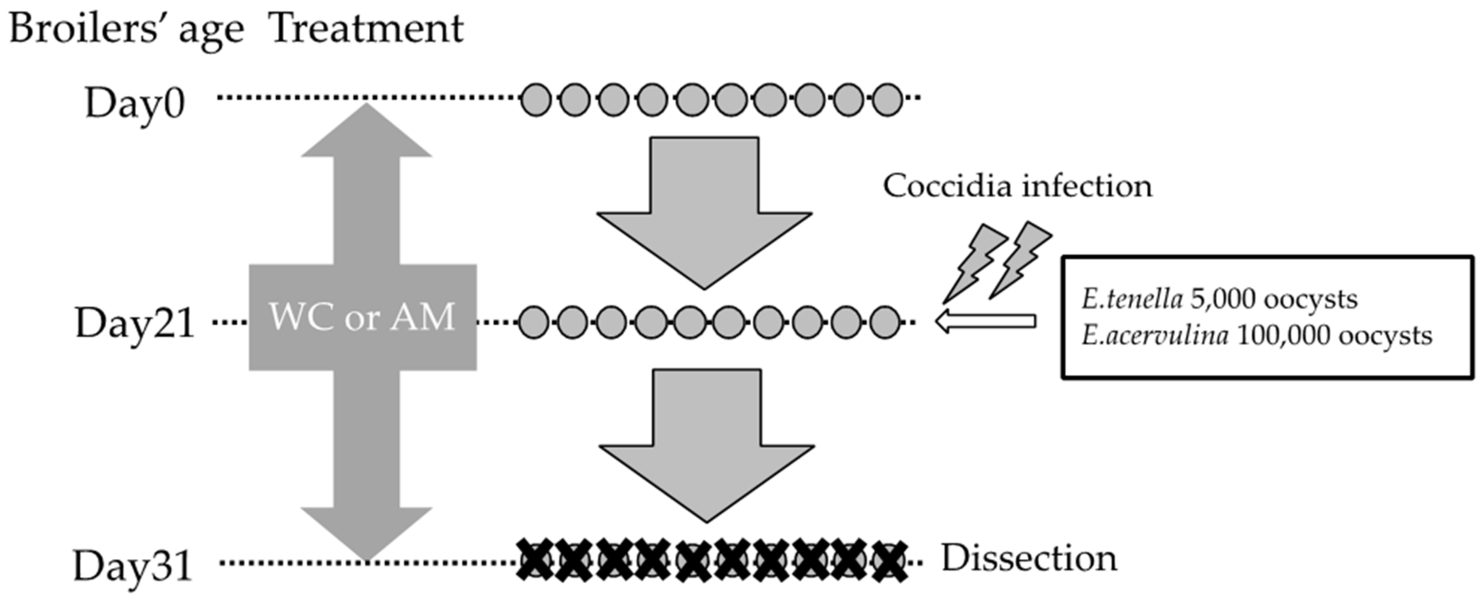

Figure S1 Experimental design and sampling procedure in Experiment 1.
Sixty chicks were purchased and divided into six groups. Each group was allocated into an individual pen. WC, *Weizmannia coagulans* strain SANK70258 supplementation; AM, lasalocid-A sodium administration. Gray circles denote individual chicks.

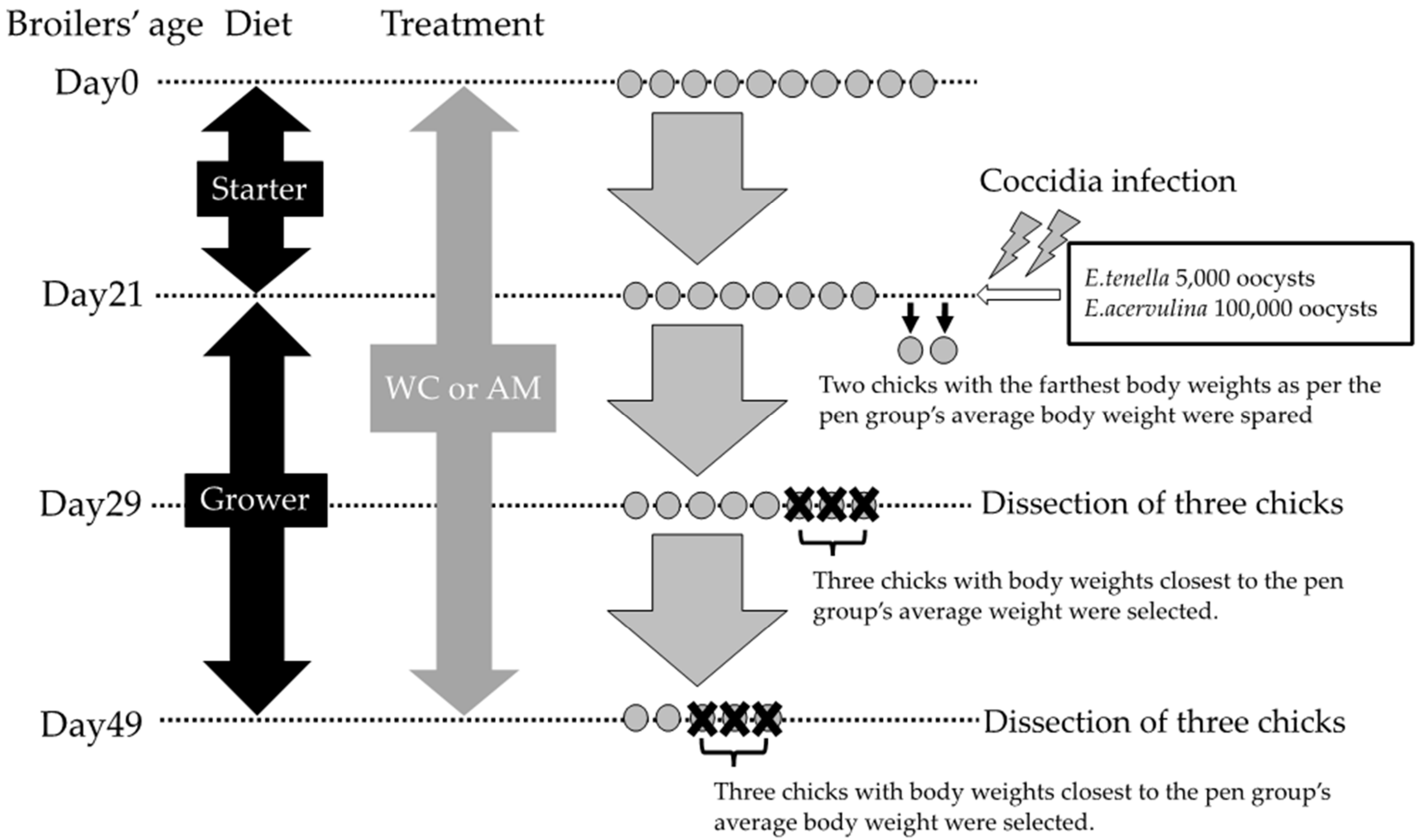

Figure S2. Experimental design and sampling procedure in Experiment 2.
Ninety chicks were purchased and divided into 9 pens. Three experimental group was designed, and each group was run in triplicate. WC, *Weizmannia coagulans* strain SANK70258 supplementation; AM, lasalocid-A sodium administration. Gray circles denote individual chicks.
